# Integrative Assessment of Left Ventricular Myocardial Work: Prognostic Utility and Clinical Application Across Cardiovascular Pathologies

**DOI:** 10.3390/jcm15031311

**Published:** 2026-02-06

**Authors:** Alexandra-Cătălina Frișan, Mihai-Andrei Lazăr, Raluca Coifan, Simina Crișan, Daniel-Miron Brie, Silvia Ana Luca, Adina Ionac, Cristian Mornoș

**Affiliations:** 1 Cardiology Department, “Victor Babeș” University of Medicine and Pharmacy, 2 Eftimie Murgu Square, 300041 Timișoara, Romania; alexandra.frisan@umft.ro (A.-C.F.); sosdean.raluca@umft.ro (R.C.);; 2 Institute of Cardiovascular Diseases Timisoara, 13A Gheorghe Adam Street, 300310 Timisoara, Romania; 3 Research Center, Institute of Cardiovascular Diseases Timisoara, 13A Gheorghe Adam Street, 300310 Timisoara, Romania

**Keywords:** myocardial work, echocardiography, heart failure, left ventricular ejection fraction, global longitudinal strain

## Abstract

Left ventricular myocardial work (MW) has emerged as a valuable echocardiographic parameter for evaluating cardiac function and predicting clinical outcomes. Unlike conventional indices such as left ventricular ejection fraction and global longitudinal strain, MW integrates myocardial deformation with left ventricular pressure, providing a load-adjusted and physiologically meaningful assessment of myocardial performance. Growing evidence demonstrates that impaired MW is consistently associated with adverse outcomes, including heart failure hospitalization, mortality, and functional deterioration, across a wide spectrum of cardiovascular conditions such as ischemic heart disease, valvular heart disease, and cardiomyopathies. The noninvasive estimation of MW using commercially available echocardiographic software has enhanced its feasibility in routine clinical practice, enabling improved risk stratification and early identification of high-risk patients. This review summarizes current evidence supporting the prognostic value of MW, highlights its incremental role beyond conventional echocardiographic parameters, and discusses future perspectives for its integration into everyday clinical decision-making.

## 1. Introduction

The accurate assessment of left ventricular (LV) systolic performance remains fundamental to cardiovascular risk stratification across a wide range of cardiac diseases. Historically, LV ejection fraction (LVEF) has served as the cornerstone metric for this purpose [1,2]. However, its well-recognized limitations—including dependency on loading conditions, reduced sensitivity to early myocardial dysfunction, and reliance on geometric assumptions—have increasingly restricted its prognostic reliability [3].

LV global longitudinal strain (GLS) has substantially improved the detection of subclinical myocardial dysfunction and the prediction of cardiovascular outcomes by identifying myocardial impairment before overt reductions in LVEF occur [4]. Nevertheless, GLS remains sensitive to afterload variations, particularly in conditions characterized by pressure overload or dynamic fluctuations in arterial load [5].

Noninvasive LV myocardial work (MW), derived from speckle-tracking echocardiography combined with brachial cuff-measured systolic blood pressure, has emerged as a promising next-generation parameter. By integrating myocardial deformation with instantaneous estimates of LV pressure, MW provides a load-adjusted and energetically meaningful quantification of myocardial performance [6]. This approach enables a more physiologically comprehensive assessment of cardiac function by accounting for both deformation and afterload. The main characteristics, limitations, and conceptual differences between GLS and MW are summarized in Table 1.

MW provides practical clinical value, particularly due to its superiority over GLS in load-dependent scenarios. Its integration into standard workflows offers rapid, physiologically meaningful insights into myocardial performance. In patients with hypertension, MW enables differentiation between acute blood pressure-related effects and chronic myocardial remodeling, refining the assessment of regional myocardial performance [8]. In coronary artery disease (CAD), MW indices like GWI identify patients at higher risk of adverse events [9]. Moreover, MW reflects altered myocardial energetics, as supported by their association with myocardial fibrosis on cardiac magnetic resonance (CMR) imaging in hypertrophic cardiomyopathy (HCM) [10]. In valvular heart disease, especially low-gradient aortic stenosis, MW helps differentiate true-severe dysfunction [11]. In cardio-oncology [12], MW refines risk stratification by detecting subclinical cardiotoxicity even when loading conditions mask GLS changes. Despite its potential, clinical interpretation must account for inter-vendor variability and the necessity of accurate, simultaneous blood pressure measurement. Emerging AI-driven automated analysis is currently addressing these limitations, enhancing reproducibility across diverse patient populations. Overall, MW serves as a robust, pressure-adjusted tool that complements LVEF and GLS, facilitating precision-based therapeutic decisions.

Despite the growing body of literature on LV MW and the availability of several reviews addressing its technical principles and applications across specific cardiovascular pathologies [6,13,14,15], the prognostic value of MW has not been the primary focus of prior syntheses. In particular, a comprehensive review integrating evidence on the prognostic implications of MW across different cardiovascular diseases and contextualizing these findings relative to established parameters such as LVEF and GLS is currently lacking. Moreover, the clinical implications of MW-derived prognostic information, including its potential role in risk stratification, therapeutic decision-making, and longitudinal patient management, have not been systematically discussed. Consequently, the extent to which MW provides consistent, incremental, and clinically actionable prognostic information across heterogeneous diseases characterized by distinct loading conditions and pathophysiological mechanisms remains insufficiently synthesized.

The purpose of this review is to synthesize current evidence regarding the prognostic role of noninvasive MW across a broad spectrum of cardiovascular conditions, including ischemic heart disease, valvular heart disease, cardiomyopathies, and heart failure (HF), and to discuss how MW-derived prognostic information may be translated into clinical practice, while highlighting emerging applications and future research directions. Selection criteria focused on original clinical research—primarily prospective and retrospective longitudinal studies—that provided incremental prognostic value of MW beyond conventional parameters (LVEF and GLS). Given the narrative framework, priority was given to high-impact studies that utilized validated noninvasive software and provided clear endpoint definitions across diverse cardiovascular phenotypes.

## 2. Principles and Physiological Basis of Myocardial Work

**Figure 1:**
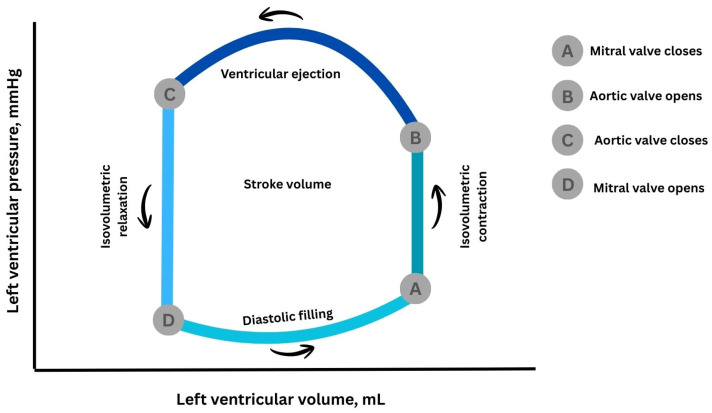
Schematic representation of the left ventricular pressure–volume loop in normal conditions. The loop illustrates the sequential mechanical events of a single cardiac cycle, including pressure rise at constant volume (isovolumetric contraction), systolic blood ejection into the arterial circulation, pressure decline at unchanged volume (isovolumetric relaxation), and diastolic ventricular filling preceding the next contraction. Different colors indicate the distinct phases of the cardiac cycle, and arrows denote their sequential progression.

At the end of the 19th century, Otto Frank conceptualized ventricular contraction using pressure–volume relationships, laying the foundation for modern cardiac mechanics [16]. To directly measure cardiac work, he created the so-called “heart indicator,” a device that could simultaneously record ventricular pressure and volume and convert these signals into orthogonal light-beam deflections captured on photographic film. Instruments that were similar in principle were used to measure the work of steam engines; the system plotted volume on one axis and pressure on the other. Adopting a coordinate layout where the vertical axis corresponds to volume and the horizontal axis to pressure, Frank was able to depict the dynamic changes in pressure and volume throughout the cardiac cycle, including filling, isovolumetric contraction, ejection, and relaxation. The resulting trace formed a closed loop in the pressure–volume plane, with the area enclosed corresponding to the heart’s external mechanical work. By also considering heart rate, Frank introduced the concept of cardiac power, representing the rate at which the heart generates mechanical work. This concept was later formalized through the pressure–volume loop (PVL), which graphically represents the cardiac cycle by plotting instantaneous ventricular pressure against volume (Figure 1). Decades later, Suga and Sagawa [17] extended Frank’s work by recording PVL in isolated canine hearts, enabling real-time characterization of ventricular energetics. They demonstrated a close correlation between the pressure–volume area—a combination of Frank’s loop and the area representing residual potential energy—and myocardial oxygen consumption, providing key insights into myocardial efficiency and energy utilization.

The later development of conductance catheter technology by Baan et al. [18] enabled in vivo assessment of pressure–volume relationships, allowing the PVL to become a cornerstone of cardiovascular physiology. The area enclosed by the PVL corresponds to stroke work and reflects the mechanical energy transferred from the myocardium to the circulation during a cardiac cycle [19]. Despite its physiological relevance, the invasive nature of PVL acquisition has limited its widespread clinical application.

Recent developments in echocardiography, particularly volumetric and strain imaging, have the enabled noninvasive evaluation of pressure–volume dynamics across the cardiac cycle. Regional MW and energy consumption can be inferred from the area of the myocardial force–segment length loop. Since directly measuring myocardial force is technically challenging, ventricular pressure is commonly used as a surrogate, and the LV pressure–dimension loop provides a means to assess regional mechanical function [20].

In 2012, Russell et al. [21] introduced a noninvasive approach to estimate MW by combining myocardial strain obtained from speckle-tracking echocardiography with an approximated LV pressure curve. To allow application across different cardiac conditions, a reference LV pressure curve was derived from invasive recordings in patients with varying contractile states. These recordings were standardized for durations of isovolumic contraction, ejection, and relaxation and scaled to consistent peak pressures. An individual patient’s LV pressure curve is then created by adjusting this reference according to valve timings measured by echocardiography and systolic blood pressure from the brachial cuff, which serves as an estimate of peak LV pressure. By maintaining the physiological link between pressure and strain, this approach enables noninvasive measurement of MW. This method produces LV pressure–strain loops (PSLs), which integrate load-adjusted pressure with regional myocardial deformation, to provide a surrogate measure of MW. The experimental part of the Russel’s study confirmed that work derived from noninvasive PSL closely corresponds to invasively measured PVL areas under a range of hemodynamic conditions [21].

**Figure 2:**
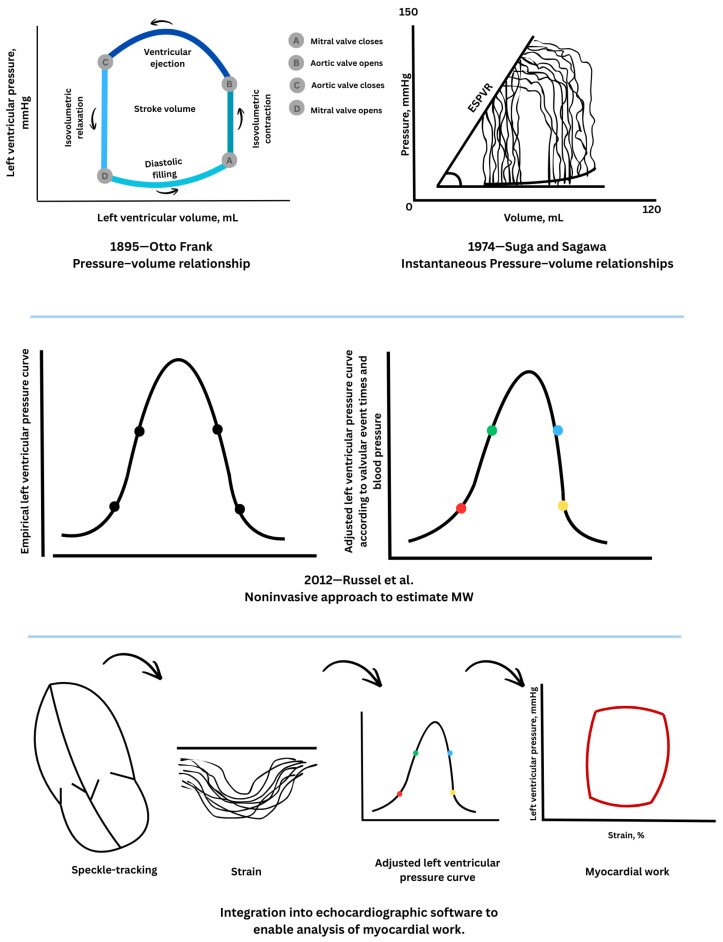
Conceptual framework and noninvasive assessment of myocardial work [16,17,18,19,20,21,22]. The figure illustrates the conceptual progression from invasive to noninvasive assessment of cardiac mechanics and myocardial work. The upper panels depict the historical development of pressure–volume analysis, beginning with Otto Frank’s description of ventricular pressure–volume relationships (1895) [16] and later advances by Suga and Sagawa (1974) [17], who introduced instantaneous pressure–volume relationships and the end-systolic pressure–volume relationship (ESPVR) to characterize ventricular energetics. The middle panels show the development of a noninvasive approach to MW estimation, based on an empirically derived left ventricular pressure curve adjusted to valvular event timing and arterial blood pressure, as proposed by Russell et al. (2012) [21]. The lower panels demonstrate the integration of speckle-tracking-derived myocardial strain with the estimated left ventricular pressure curve to generate pressure–strain loops, whose enclosed area provides a surrogate measure of regional myocardial work. This framework preserves the physiological coupling between loading conditions and myocardial deformation while enabling routine, noninvasive clinical application. Colors and dots indicate corresponding phases of the cardiac cycle and key valvular events (mitral and aortic valve opening and closure), while arrows in the lower panel illustrates the methodological workflow for integrating deformation and pressure data to estimate myocardial work.

Today, this technique is integrated into dedicated echocardiographic software, facilitating its routine application in clinical practice [22]. A schematic overview of the physiological principles and historical progression of MW assessment is presented in Figure 2.

## 3. Noninvasive Echocardiographic Measurement of Myocardial Work

In current echocardiographic platforms, MW analysis is performed after acquisition of GLS from standard apical two-, three-, and four-chamber views. To ensure reliable tracking, high-quality images with a frame rate exceeding 40 frames per second are required. Acquisition of at least three consecutive cardiac cycles per view is recommended, and particular care must be taken to avoid apical foreshortening, which may lead to overestimation of MW [15].

Brachial blood pressure should be measured immediately before or during image acquisition, with the patient maintained in the same position (typically left lateral decubitus). Valvular event timing is determined using pulsed-wave Doppler interrogation of the mitral and aortic valves and confirmed in the apical long-axis view [13]. Data are subsequently analyzed offline using dedicated software (e.g., EchoPAC, GE Healthcare, version 206), which generates pressure–strain loops and derived MW indices [22].

Following data input, a MW bull’s-eye plot is generated, analogous to GLS displays. Four principal parameters are derived [13,14,15]:

- Global work index (GWI, mmHg%): represents the total work performed by the LV during systole, calculated from mitral valve closure to mitral valve opening, encompassing both isovolumic and ejection phases.
- Global constructive work (GCW, mmHg%): reflects effective MW contributing to LV ejection, defined as myocardial shortening during systole and lengthening during isovolumic relaxation.
- Global wasted work (GWW, mmHg%): represents inefficient energy expenditure resulting from myocardial lengthening during systole and shortening during isovolumic relaxation.
- Global work efficiency (GWE, %): calculated as GCW divided by the sum of GCW and GWW, representing the proportion of myocardial energy converted into effective cardiac output.

Global MW indices are computed as the average of all LV segments, while regional values can be assessed individually. In the bull’s-eye display, green indicates normal work, blue represents wasted work, and red denotes regions of increased workload [22].

**Figure 3:**
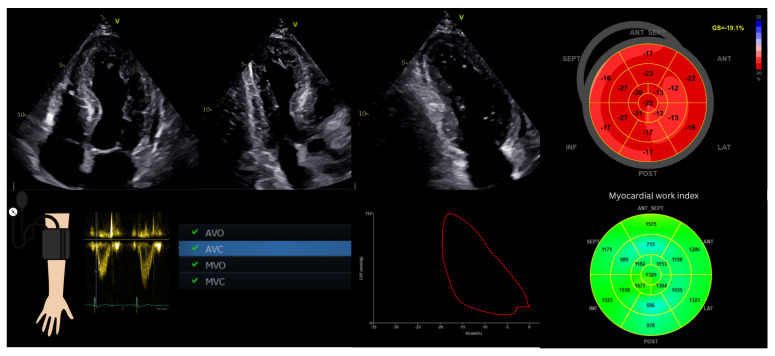
Myocardial work assessment includes two-dimensional grayscale imaging in apical two-, three-, and four-chamber views, followed by global longitudinal strain and left ventricular bull’s-eye analysis. Activation of the myocardial work module allows input of brachial blood pressure and determination of valvular timing. The output includes regional and global myocardial work indices, pressure–strain loops, and derived parameters such as global constructive and wasted work. ANT_SEPT, antero-septal; ANT, anterior; LAT, lateral; POST, posterior; INF, inferior; SEPT, septal; GS, global strain; AVO, aortic valve opening; AVC, aortic valve closure; MVO, mitral valve opening; MVC, mitral valve closure; V, apical view orientation marker.

Figure 3 illustrates the steps of MW acquisition.

Table 2 summarizes the normal reference ranges for healthy individuals, as reported in the EACVI NORRE study (European Association of Cardiovascular Imaging Normal Reference Ranges for Echocardiography) [23].

## 4. Methodological Limitations in Myocardial Work Measurement

Although MW offers a physiologically appealing framework for evaluating LV performance, several methodological limitations must be acknowledged when interpreting its clinical applicability.

MW analysis is inherently dependent on the quality of speckle-tracking-derived strain measurements and therefore shares many of the technical limitations of GLS. Suboptimal acoustic windows, inadequate endocardial border delineation, and insufficient temporal resolution can compromise tracking accuracy and result in unreliable MW estimation. A frame rate of at least 40 frames per second is generally required to ensure adequate temporal resolution, while arrhythmias—particularly atrial fibrillation or frequent ectopy—may preclude reliable averaging of cardiac cycles and reduce measurement reproducibility [6].

Accurate noninvasive estimation of LV pressure represents another critical determinant of MW reliability. The method assumes that brachial systolic blood pressure accurately reflects peak LV systolic pressure, provided that measurements are obtained contemporaneously and in the same body position as image acquisition [13]. Any discrepancy in blood pressure measurement can significantly influence MW indices, particularly in patients with labile hemodynamics.

At present, MW assessment is available only through vendor-specific software platforms, limiting cross-platform reproducibility and standardization. This lack of vendor neutrality remains a significant barrier to widespread clinical adoption and multicenter comparability.

Pathophysiological conditions may further compromise MW accuracy. In the presence of severe aortic stenosis, LV systolic pressure exceeds peripheral arterial pressure, resulting in systematic underestimation of MW if uncorrected. To address this limitation, recent studies have proposed adding the mean transvalvular gradient to the systolic blood pressure, which has demonstrated strong agreement with invasive measurements [24]. Similarly, in the presence of significant mitral regurgitation, interpretation of MW becomes complex, as effective forward stroke volume does not fully reflect myocardial energy expenditure [13].

Additional theoretical limitations include the inability of current MW models to account for myocardial wall stress, regional wall thickness, or ventricular curvature, all of which influence myocardial workload. Moreover, diastolic pressure dynamics are not incorporated into pressure–strain loops, potentially affecting MW interpretation in conditions associated with elevated filling pressures [14]. Taken together, these factors underscore the importance of the contextual interpretation of MW parameters and reinforce the need for disease-specific validation.

## 5. Prognostic Implications of MW in Coronary Artery Disease

Risk stratification in coronary artery disease has traditionally relied on global systolic indices such as LVEF and, more recently, GLS [5]. However, these parameters remain influenced by loading conditions and may fail to detect subtle ischemia-related myocardial dysfunction, particularly in patients with preserved ejection fraction or multivessel disease [4]. By integrating myocardial deformation with afterload, MW provides a more physiologically comprehensive assessment of LV performance. Growing evidence supports the prognostic value of MW in both stable and acute coronary syndromes, enabling earlier identification of subclinical dysfunction and improved prediction of adverse cardiovascular outcomes.

### 5.1. Chronic Coronary Syndromes

Contemporary understanding of chronic coronary syndromes (CCSs) extends beyond obstructive CAD to include structural and functional abnormalities affecting both the epicardial and microvascular coronary circulation. Transient ischaemia may result from diffuse atherosclerosis, dynamic epicardial processes such as vasospasm or myocardial bridging, congenital coronary anomalies, or coronary microvascular dysfunction, even in the absence of flow-limiting stenoses. In addition, extracoronary factors—including anemia, tachycardia, blood pressure fluctuations, myocardial hypertrophy, and fibrosis—can further contribute to the multifactorial mechanisms underlying chronic myocardial ischaemia. In this complex setting, echocardiography plays a central role by identifying subtle functional alterations despite preserved LVEF, through the detection of regional wall motion abnormalities, myocardial deformation disturbances, and alterations in MW [25].

Early identification of myocardial dysfunction in CCS is crucial for both diagnostic evaluation and risk stratification. Growing evidence suggests that MW indices provide incremental diagnostic and prognostic information beyond conventional echocardiographic parameters.

In a single-center observational study of 115 patients with preserved LVEF and no resting wall motion abnormalities, noninvasive MW at rest was significantly reduced in those with angiographically significant CAD. Global MW showed superior diagnostic performance compared with GLS, particularly in detecting single-vessel disease. By integrating myocardial deformation with afterload, MW appears more sensitive to early ischemia-related energetic impairment, supporting its potential role as a resting, noninvasive tool for the early identification of clinically significant CAD [9].

Sabatino et al. [26] further demonstrated that regional MW indices—particularly regional GWE—provide high diagnostic accuracy for detecting critical coronary artery stenosis. In patients with angiographically significant CAD, global MW indices were reduced despite preserved systolic function, while regional MW efficiency was selectively impaired in myocardial segments subtended by the stenotic vessel. Notably, regional MW efficiency outperformed longitudinal strain and post-systolic shortening in identifying ischemic territories, underscoring the clinical value of MW for early, noninvasive detection of functionally significant CAD prior to invasive angiography. Similarly, in patients with angina and non-obstructive CAD, resting myocardial work indices—particularly global and regional MW efficiency—were strongly associated with impaired stress myocardial perfusion assessed by computer tomography myocardial perfusion imaging, outperforming GLS and enabling the identification of microvascular ischemia despite the absence of epicardial stenoses [27].

In a cohort of 131 patients with clinically diagnosed stable CAD, preserved LVEF, and no resting wall motion abnormalities, MW analysis demonstrated significant value in identifying individuals at high ischemic risk. Both GWI and GCW showed good discriminatory ability for high-risk stable CAD, with clinically meaningful cutoff values and independent associations with disease severity after multivariable adjustment. Importantly, regional MW index was selectively reduced in coronary territories subtended by significantly stenotic vessels, and combining regional MW impairment across multiple perfusion territories further improved diagnostic performance. These findings support the role of global and regional MW as sensitive, noninvasive markers for the early identification of high-risk stable CAD in patients with preserved systolic function, potentially aiding in risk stratification and the selection of patients who may benefit from timely revascularization [28].

Furthermore, the clinical utility of MW in CCS extends beyond diagnosis and risk stratification to the assessment of functional capacity and therapeutic response. Recent data in patients undergoing cardiac rehabilitation after coronary artery bypass grafting have demonstrated significant associations between MW indices and exercise tolerance, including performance on the six-minute walk test, supporting the role of MW as a marker of functional recovery and cardiovascular efficiency [29]. These findings highlight the potential of MW to provide a comprehensive evaluation of myocardial performance in CCS.

### 5.2. Acute Coronary Syndromes

A growing body of evidence supports the prognostic utility of MW in patients with acute coronary syndromes, particularly following ST-segment elevation myocardial infarction (STEMI).

In a retrospective cohort study of 507 STEMI patients, Lustosa et al. [30] showed that reduced GWE (<86%) independently predicted a higher risk of long-term all-cause mortality during a median follow-up of 80 months, offering prognostic information beyond LVEF. In their study, MW analysis was performed within 48 h of hospital admission. Similarly, Coisne et al. [31] showed that, in 244 patients hospitalized for acute myocardial infarction, GWE measured by transthoracic echocardiography one month after the event independently predicted major adverse cardiovascular events (MACEs), over a median follow-up of 681 days. A GWE threshold of <91% identified patients at nearly a threefold higher risk, highlighting its incremental value for post-acute myocardial infarction risk stratification.

Wang et al. [32] further demonstrated that, in patients with first anterior STEMI treated with primary percutaneous coronary intervention (PCI), early LV remodeling (≥20% increase in LV end-diastolic volume at 3 months) occurred in 33% of patients and was associated with a lower global and culprit-region MW index, constructive work, and work efficiency, as well as greater acute-phase differences between culprit and non-culprit regions. Inter-territorial differences in GWE independently predicted adverse remodeling, highlighting the prognostic value of inhomogeneous MW distribution after anterior STEMI. Butcher et al. [33] investigated 197 STEMI patients with reduced LVEF (≤40%) treated with primary PCI. Baseline GWI was independently associated with both functional recovery and long-term prognosis. Higher baseline GWI predicted LVEF normalization at 6 months, while lower GWI (<750 mmHg%) was independently associated with increased all-cause mortality over a median follow-up of 112 months, providing prognostic information incremental to LVEF and GLS.

In a separate cohort, Timóteo et al. [34] reported that reduced GWI, measured by echocardiography 5 ± 3 days post-admission, independently predicted medium-term adverse cardiovascular outcomes—including cardiovascular death, nonfatal myocardial infarction, and unplanned cardiovascular admissions—over a mean follow-up of 790 days. In multivariable analysis, lower GWI was the only independent predictor of events, with a threshold of ≤1165 mmHg% identifying higher risk, highlighting the incremental prognostic value of MW over traditional risk factors and ejection fraction (EF). Additionally, Sun et al. [35] investigated 112 STEMI patients treated with primary PCI. Segmental MW and microvascular perfusion were assessed using noninvasive PSL and myocardial contrast echocardiography, respectively. Segmental MW indices correlated with microvascular perfusion, and both were independently associated with segmental LV recovery at 3 months. Combining MW efficiency and microvascular perfusion improved prediction of segmental recovery compared with either measure alone. During a median follow-up of 42 months, regional MW parameters were also associated with long-term cardiac events, demonstrating that segmental MW provides incremental prognostic value in reperfused STEMI.

**Figure 4:**
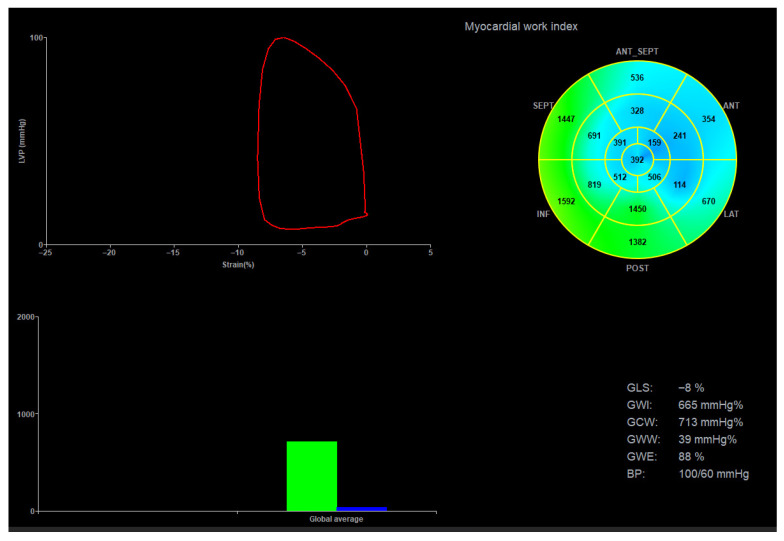
Myocardial work analysis in a patient with acute anterior myocardial infarction. Global reductions in GWI, GCW and GWE, together with impaired GLS, indicate marked myocardial dysfunction. These findings suggest a higher risk of adverse outcomes and highlight the potential of MW indices to provide additional prognostic information beyond conventional measures. GLS, global longitudinal strain; EF, ejection fraction; GWI, global work index; GCW, global constructive work; GWW, global wasted work; GWE, global work efficiency; BP, blood pressure. Red line represents the left ventricular pressure–strain loop. In the bull’s-eye plot, green indicates normal myocardial work, while blue highlights areas of negative work in the anteroseptal segments, suggesting impaired myocardial function in those regions.

Meimoun et al. [36] further showed that, in patients with anterior STEMI, GCW outperformed other MW indices and GLS in predicting both segmental and global LV recovery after PCI. Additionally, GCW was more impaired in patients experiencing in-hospital complications. These findings suggest that GCW provides incremental prognostic value for identifying both functional recovery and early adverse events after anterior STEMI. Figure 4 shows MW analysis in a patient with acute anterior myocardial infarction.

## 6. Prognostic Implications of MW in Patients with Mitral Valve Disease

**Figure 5:**
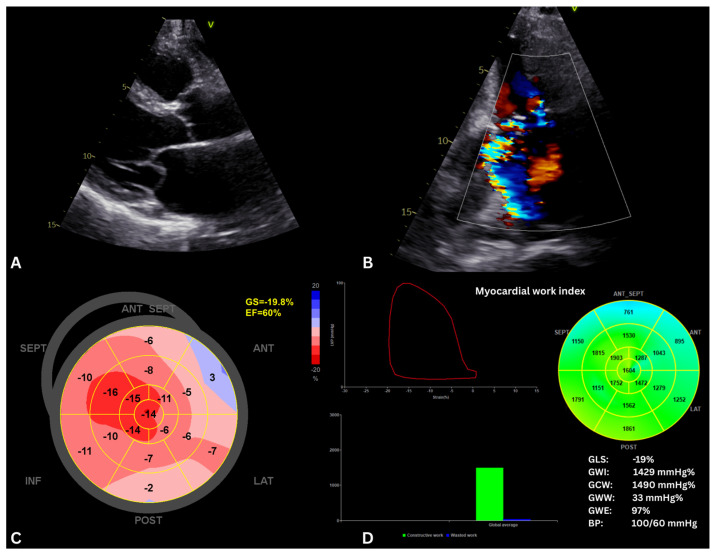
Myocardial work analysis in a patient with severe mitral regurgitation. (**A**) Parasternal long-axis view showing posterior mitral valve prolapse. (**B**) Apical 2-chamber view with color Doppler demonstrating severe mitral regurgitation with posteriorly directed jet. (**C**) Speckle-tracking analysis identifying preserved LV function (GLS −19.8%, LVEF 60%). (**D**) Myocardial work analysis showing preserved GWI, GCW and GWE, but very low wasted work (GWW = 33 mmHg%), suggesting minimal energy loss. Patients with extremely low GWW, even with preserved LVEF and GLS, may be at higher risk of adverse outcomes [38], including progression of heart failure or complications from severe mitral regurgitation. In this context, MW could help identify patients who might benefit from earlier or more aggressive management, such as closer follow-up, optimization of afterload-reducing therapy, or consideration for mitral valve intervention. In the panel (**C**), red shades indicate negative strain values, while blue indicates positive strain values. In panel (**D**), the myocardial work bull’s-eye shows green for normal/constructive myocardial work and blue for negative/wasted work. GS/GLS, global longitudinal strain; EF, ejection fraction; GWI, global work index; GCW, global constructive work; GWW, global wasted work; GWE, global work efficiency; BP, blood pressure; V, apical view orientation marker.

Assessment of LV function in mitral regurgitation (MR) remains clinically challenging, as traditional indices such as LVEF and GLS are heavily influenced by altered loading conditions and may mask early myocardial dysfunction [37]. By accounting for afterload, MW provides a more physiologically meaningful evaluation of LV performance in this population (Figure 5).

Yedidya et al. [38] demonstrated that reduced GWI and GCW were independently associated with increased all-cause mortality in patients with secondary MR, whereas LVEF showed no prognostic significance. Interestingly, lower GWW (<300 mmHg%)—reflecting reduced wasted energy—was paradoxically associated with worse outcomes, likely reflecting an inability of the LV to generate sufficient work to overcome systemic afterload.

In patients with secondary mitral regurgitation undergoing transcatheter edge-to-edge repair (TEER), LV MW indices were investigated for their association with forward flow reserve, measured as forward stroke volume index. Seventy patients were evaluated at baseline and 6 months post-TEER and divided into improvers and non-improvers. After TEER, forward stroke volume index improved overall, while non-improvers showed a significant decline in LV GLS, GWI, and GCW, indicating worsening LV systolic function. In contrast, these parameters remained stable in improvers. Baseline GWE remained the only independent predictor of forward stroke volume index after multivariable adjustment [39].

In 181 HF patients with functional MR and LVEF < 50%, LV function was assessed using GLS and GWI, with a median follow-up of 42 months for cardiovascular events. While several echocardiographic parameters were associated with events in univariate analysis, only GLS and GWI remained independent predictors after multivariate adjustment. GWI provided no incremental prognostic value over GLS [40].

In a cohort of 306 patients who underwent surgical repair for primary MR, preoperative assessment of MW revealed that impaired GWI was significantly associated with increased long-term mortality over a median follow-up of 5 years. Importantly, GWI provided incremental prognostic value beyond GLS, suggesting that MW assessment may offer additional insight into risk stratification prior to mitral valve surgery [41]. Moreover, in a retrospective analysis of 58 high-surgical-risk patients with moderate-to-severe or severe functional MR undergoing percutaneous edge-to-edge mitral valve repair, echocardiographic parameters including LVEF, GLS, and MW were assessed at baseline and 1 year post-MitraClip implantation. MR severity significantly decreased, while LVEF and GLS remained largely unchanged. In contrast, GWI and GCW improved significantly, reflecting enhanced LV performance. Notably, baseline GCW was the only parameter significantly associated with LV end-systolic volume reduction. These findings suggest that preserved baseline GCW may serve as a strong predictor of LV reverse remodeling after MitraClip implantation [42].

## 7. Prognostic Implications of MW in Aortic Stenosis and After Transcatheter Aortic Valve Implantation

In AS, chronic pressure overload induces adaptive hypertrophy that may preserve LVEF despite progressive myocardial dysfunction. Because MW incorporates afterload, it offers a more physiologically appropriate assessment of LV performance in this context. Importantly, LV systolic pressure in AS exceeds brachial systolic pressure, necessitating correction by adding the mean transvalvular gradient [11].

In a cohort of 170 asymptomatic patients with moderate-to-severe AS and preserved LVEF, noninvasive MW indices were evaluated for their relationship with stages of cardiac damage and prognostic value. Over a mean follow-up of 30 months, 27 patients died, and multivariable analysis showed that lower GWI and GCW were independently associated with all-cause and cardiovascular mortality. Thresholds of GWI ≤ 1951 mmHg% and GCW ≤ 2475 mmHg% predicted long-term death, suggesting that MW indices can help identify asymptomatic AS patients at higher risk despite preserved LVEF [43]. In patients with moderate AS, however, a GWI ≤ 1951 mmHg% did not reliably predict the need for valve replacement, underscoring the importance of disease-stage-specific interpretation [44].

In bicuspid aortic valve patients with severe AS undergoing surgical valve replacement, reduced LV MW—specifically lower GWI and GCW—was independently associated with early postoperative major cardiovascular events, outperforming LV GLS and highlighting MW as a sensitive tool for detecting subclinical LV dysfunction and optimizing intervention timing [45].

**Figure 6:**
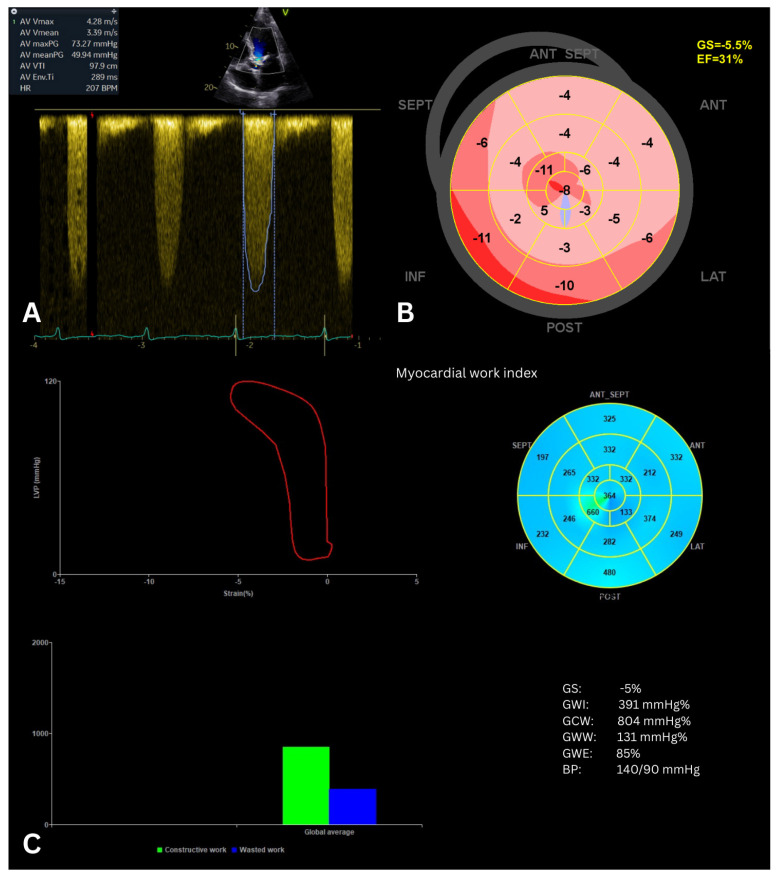
Echocardiographic assessment of a patient with severe aortic stenosis before TAVR. (**A**) Continuous-wave Doppler across the aortic valve demonstrates high velocities consistent with elevated gradients. (**B**) Bullseye plot from speckle-tracking shows severely reduced GLS (−5.5%) and LVEF (31%), indicating profound systolic impairment. (**C**) Myocardial work analysis reveals markedly reduced GWI and GCW, with the bullseye predominantly blue, indicating minimal or negative work across all LV segments. These results highlight limited contractile LV reserve, suggesting high risk for adverse outcomes. The right-shifted pressure-strain loop (red line) in this patient essentially reflects impaired contractility under high afterload. GS, global longitudinal strain; EF, ejection fraction; GWI, global work index; GCW, global constructive work; GWW, global wasted work; GWE, global work efficiency; BP, blood pressure; V, apical view orientation marker.

Recent studies consistently demonstrate that MW indices, particularly GWI, provide valuable insights into LV function and prognosis in patients with severe AS treated with transcatheter aortic valve replacement (TAVR). In a large cohort of 255 patients, LV MW indices such as GWI, GCW, and GWE change significantly after TAVR, and post-TAVR GWI showed the strongest independent association with all-cause mortality, outperforming other LV function measures in prognostic models [46]. Another study confirmed that lower values of baseline GWI are independently associated with long-term mortality after TAVR, with better predictive value than LVEF, GLS, and other MW indices [47]. Subgroup analyses across AS hemodynamic categories revealed that higher pre-TAVR GWI predicts reduced mortality irrespective of flow/gradient pattern, while GWI’s post-procedure change varies by baseline LV function [48]. Finally, baseline risk stratification research showed that a lower baseline GWI (<2323 mmHg%) is linked with increased mortality and heart failure hospitalization after TAVR, and adding GWI to risk models significantly improves outcome prediction [49]. Overall, MW assessment—especially GWI—emerges as a promising echocardiographic marker for assessing LV function and improving risk stratification in severe AS patients undergoing TAVR, potentially enhancing clinical decision-making beyond traditional measures. Figure 6 illustrates the echocardiographic assessment of a patient with severe AS.

## 8. Prognostic Implications of MW for Identification of Cancer Therapy-Related Cardiotoxicity

GLS is currently recommended for the surveillance of cancer therapy-related cardiac dysfunction (CTRCD) [50]. However, GLS is susceptible to changes in loading conditions, which are common during oncologic treatment.

In women with HER2+ breast cancer undergoing anthracycline and trastuzumab therapy, changes in echocardiographic GLS and MW indices, particularly GWI and GCW, were associated with CTRCD diagnosed by cardiac magnetic resonance. Absolute changes in GLS and GWI correlated with concurrent CTRCD, while changes in GLS, GWI, and GCW predicted CTRCD at subsequent visits. MW indices provided incremental diagnostic value over GLS in selected cases, especially in patients with minimal GLS changes but substantial systolic blood pressure reductions. However, MW indices did not enhance prognostic prediction beyond GLS for future CTRCD. These findings suggest that GLS remains the primary tool for CTRCD detection and risk stratification, with MW offering complementary information in specific scenarios [51].

## 9. Prognostic Implications of MW in Patients with Cardiomyopathies

### 9.1. Hypertrophic Cardiomyopathy

Hypertrophic cardiomyopathy (HCM) is characterized by early myocardial dysfunction driven by myocyte disarray, fibrosis, and microvascular ischemia [52,53]. Strain abnormalities often precede reductions in LVEF and correlate with fibrosis burden and arrhythmic risk [54].

In 82 patients with non-obstructive HCM, GCW was significantly reduced compared to age-matched healthy controls, despite preserved LVEF. GCW was the only independent predictor of LV fibrosis assessed by late gadolinium enhancement on CMR, with a cutoff of 1623 mmHg%. These findings suggest that GCW provides a sensitive measure of myocardial performance and may serve as a noninvasive marker of fibrosis in HCM, identifying subclinical myocardial impairment not detectable by conventional echocardiographic parameters [55]. Consistent results from a smaller cohort showed that a GCW threshold ≤ 1550 mmHg% was associated with extensive fibrosis on CMR, outperforming GLS [10]. Furthermore, reduced GCW correlated with wall thickness, diastolic dysfunction, and electrical abnormalities in patients with non-obstructive HCM, while higher GCW values were associated with better event-free survival, indicating that MW not only reflects LV functional impairment but also provides prognostic information in HCM [56]. In HCM pediatric populations, MW deteriorates over time and correlates with adverse events, although it does not yet outperform GLS prognostically [57].

**Figure 7:**
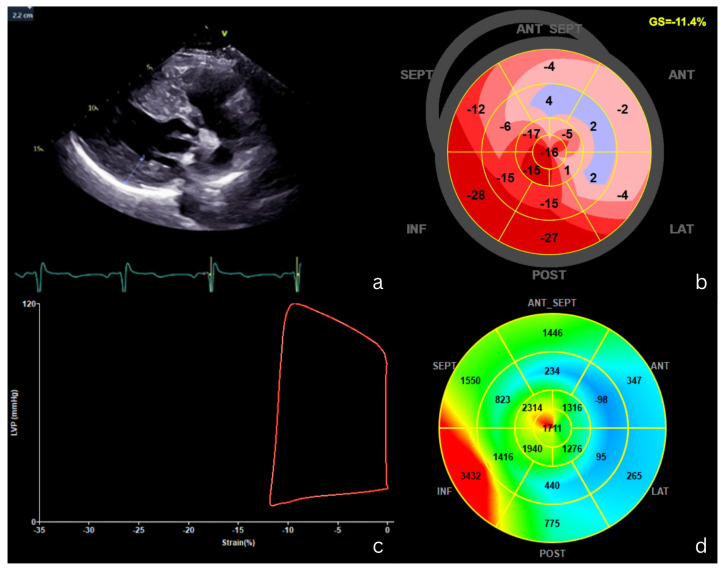
MW analysis in a patient with hypertrophic cardiomyopathy. (**a**) Parasternal long-axis view demonstrates severe ventricular hypertrophy. (**b**) Global longitudinal strain bull’s-eye plot shows impaired longitudinal deformation in hypertrophied antero-lateral segments, with a GLS of −11.4%. (**c**) The pressure–strain loop displays a reduced enclosed area and a rightward shift, indicating marked impairment of myocardial work. (**d**) The myocardial work index bull’s-eye plot demonstrates reduced myocardial work in the basal antero-lateral segment (light-blue shaded) and increased work in the inferior segment (red shaded). These findings indicate significant regional myocardial dysfunction and heterogeneity, which are associated with higher risk of adverse clinical events, highlighting the potential prognostic value of MW indices in hypertrophic cardiomyopathy. GS, global longitudinal strain; ANT, anterior; LAT, lateral; POST, posterior; ANT SEPT, antero-septal; INF, inferior; SEPT, septal; V, apical view orientation marker.

Figure 7 illustrates the MW analysis in a patient with hypertrophic cardiomyopathy.

### 9.2. Dilated Cardiomyopathy

In a retrospective analysis of 116 patients with dilated cardiomyopathy (DCM), MW indices, specifically GWI and GCW, independently predicted major adverse cardiac events over a mean follow-up of 5.1 years. Patients with GWI < 788 mmHg% or GCW < 1238 mmHg% had significantly higher risk of major adverse events. MW provided incremental prognostic value beyond conventional measures such as LVEF and GLS, suggesting it is a valuable tool for risk stratification in DCM [58].

### 9.3. Restrictive Cardiomyopathy

In restrictive cardiomyopathy, MW parameters are significantly altered despite preserved LVEF. Li et al. demonstrated that GWI, GCW, and GWE were reduced, whereas GWW was increased in patients with advanced disease, reflecting inefficient myocardial mechanics. These parameters were independently associated with adverse clinical outcomes [59].

Cardiac amyloidosis is characterized by progressive myocardial infiltration leading to restrictive physiology and systolic impairment [60]. MW has emerged as a robust prognostic marker in this population. In a prospective multicenter cohort of 100 cardiac amyloidosis patients, comprehensive echocardiography was performed and patients were followed for a median of 490 days to assess major adverse cardiac events and all-cause mortality. In this context, reduced GWI and altered apical-to-basal work distribution independently predicted major events and all-cause mortality, outperforming GLS [61]. Among 70 patients with cardiac amyloidosis followed for a median of 16 months, over half experienced the combined endpoint of all-cause mortality or HF readmission. Patients with events exhibited significantly more impaired systolic function, with lower LVEF, worse LVGLS and markedly reduced GCW. On multivariable analysis, LVEF, LVGLS, and GCW remained independent predictors of outcome; however, the model incorporating GCW showed the highest prognostic discrimination. A GCW threshold of <1443 mmHg% predicted adverse outcomes with high sensitivity (94%) and moderate specificity (64%), supporting myocardial work—particularly GCW—as a robust and incremental prognostic marker beyond conventional echocardiographic indices in cardiac amyloidosis [62].

Across three independent cohorts of patients with light-chain cardiac amyloidosis, MW indices consistently demonstrated strong short-term prognostic value. Reduced GWI and GCW were associated with higher risks of mortality and adverse cardiac events, outperforming conventional echocardiographic parameters. MW indices provided incremental prognostic information beyond established staging systems and standard imaging markers, improving risk stratification in the early disease course. Furthermore, longitudinal assessment revealed that improvements in MW following anti-plasma cell therapy were associated with improved survival, supporting MW as a dynamic marker of treatment response. Collectively, these studies highlight MW analysis as a robust, treatment-sensitive, and clinically meaningful tool for prognostic assessment in light-chain cardiac amyloidosis [63,64,65]. Similar findings have been reported in transthyretin amyloidosis, where MW indices progressively deteriorate over time and independently predict mortality and HF hospitalization [66].

## 10. Prognostic Implications of MW in Heart Failure

In HF, both LVEF and GLS are established prognostic markers [4]. However, MW has emerged as a powerful complementary tool. In a large cohort of 508 HF with reduced EF patients, reduced GWI was independently associated with a higher risk of all-cause mortality and HF hospitalization over 1 year, even after adjustment for clinical and echocardiographic variables. Notably, when GWI was included in multivariable models, neither ejection fraction nor GLS remained independent predictors, underscoring the incremental value of MW. Patients with markedly reduced GWI (<750 mmHg%) had a more than threefold increased risk of adverse outcomes, highlighting MW as a powerful tool for risk stratification in HF with reduced EF [67].

In patients with advanced HF, MW assessment is feasible and provides clinically relevant prognostic information. In this cohort, LV MW indices—GWI and GCW—were not associated with the composite outcome of death, heart transplantation, or LV assist device implantation, but were independently predictive of unplanned HF hospitalization. Higher GWI and GCW were associated with reduced hospitalization risk, and a GWI threshold identified patients with better event-free survival. These findings suggest that MW parameters may refine risk stratification in advanced HF by identifying patients at higher risk of recurrent decompensation who may benefit from closer surveillance or intensified therapy [68]. GWI also correlates with established prognostic markers, including exercise capacity and natriuretic peptide levels, with values below 500 mmHg% identifying patients with severe disease [69].

In patients hospitalized for acute HF, echocardiography-derived MW indices capture dynamic myocardial responses to decompensation and recompensation that are not reflected by LVEF. GCW and GWI improved in HF reduced EF patients who experienced biochemical recovery, while GWW remained unchanged or increased in those with persistent or worsening congestion, particularly in HF preserved EF. Importantly, elevated GWW independently predicted 6-month mortality or rehospitalization, highlighting its potential as a load-independent prognostic marker across HF phenotypes. These findings support MW assessment as a sensitive tool for monitoring therapy response and short-term risk stratification in acute HF [70].

**Figure 8:**
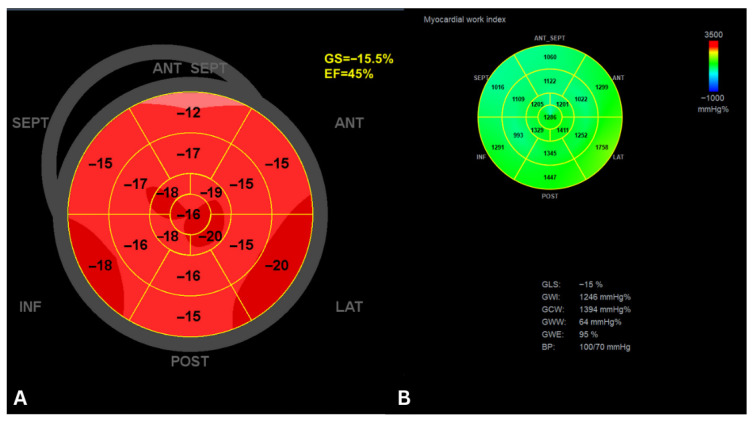
Myocardial work analysis in a patient with heart failure and mildly reduced EF. (**A**) Bullseye plot from speckle-tracking echocardiography showing a GLS of −15.5%, indicating moderately reduced systolic function. (**B**) Myocardial work analysis plot indicates largely preserved contractile efficiency with minimal wasted work. GWI has a value of 1246 mmHg%, indicating a reduced risk of hospitalization for heart failure. GS, global longitudinal strain; ANT, anterior; LAT, lateral; POST, posterior; ANT SEPT, antero-septal, INF, inferior; SEPT, septal.

In HF with mildly reduced EF, MW indices—particularly the GWI—emerged as an independent predictor of HF hospitalization. In a cohort of 273 patients followed for a median of 31 months, higher GWI and GCW were associated with lower hospitalization risk, outperforming traditional measures like LVEF and GLS. Patients with GWI ≥ 850 mmHg% had a markedly reduced risk of HF hospitalization, highlighting MW as a sensitive and clinically meaningful marker for risk stratification in HF mildly reduced EF [71]. A representative example of the analysis of MW in a patient with HF and mildly reduced LVEF is shown in Figure 8.

In HF with preserved EF and preclinical diastolic dysfunction, noninvasive MW emerged as a sensitive predictor of adverse outcomes. GWW accurately predicted first HF hospitalization, while GCW identified patients at risk of subsequent functional deterioration [72,73].

## 11. Implications for Clinical Practice

Building on the disease-specific prognostic evidence summarized above, MW provides practical clinical value across a wide range of cardiovascular conditions. Its noninvasive nature and integration with standard strain analysis make MW readily applicable in routine practice, offering rapid, physiologically meaningful insights into myocardial performance.

In CAD, MW identifies patients at higher risk of adverse events, supporting individualized management and early intervention. In valvular heart disease, preoperative assessment of MW can predict postoperative outcomes and help guide the optimal timing of interventions. In cardio-oncology, MW refines risk stratification by detecting patients at increased risk of cardiac dysfunction even when GLS changes are modest or affected by loading conditions. In cardiomyopathies and HF, MW enables early identification of myocardial dysfunction, monitors disease progression, and provides incremental prognostic information beyond conventional measures such as LVEF and GLS, facilitating tailored therapeutic decisions.

The interpretation of MW prognostic data must account for inherent methodological heterogeneity. Discrepancies between findings may arise from inter-vendor variability (e.g., GE EchoPAC vs. Philips QLAB), as software-specific algorithms for pressure curve estimation and cardiac cycle timing can influence absolute MW values. Furthermore, the accuracy of brachial cuff sphygmomanometer as a surrogate for peak systolic ventricular pressure remains a critical variable, particularly in patients with peripheral arterial disease or significant transvalvular gradients. Beyond technical factors, clinical heterogeneity—driven by diverse patient comorbidities, variations in afterload states, and different follow-up durations—requires a nuanced interpretation of “normal” vs. “pathological” MW thresholds. Future efforts towards universal software calibration and AI-assisted blood pressure integration are essential to harmonize prognostic cutoffs across clinical centers.

Overall, MW represents a versatile and sensitive tool that complements existing echocardiographic parameters, enhances risk stratification, and supports patient-centered care by translating prognostic insights into actionable clinical decisions across diverse cardiovascular conditions.

A summary of MW indices, their incremental prognostic value, and key clinical considerations across major cardiovascular conditions is provided in Table 3.

## 12. Future Directions

Future research should focus on large, prospective, multicenter studies to validate the incremental prognostic value of MW beyond established echocardiographic markers. Beyond risk stratification, the trajectory of MW indices is moving towards integrated precision phenotyping. Future research must prioritize the validation of MW in monitoring response to novel pharmacological therapies, such as SGLT2 inhibitors and GLP-1 receptor agonists, where subclinical energetic improvements may precede structural remodeling. Furthermore, the diagnostic horizon of MW includes its application in multimodal imaging fusion, combining the temporal resolution of echocardiographic pressure–strain loops with the tissue characterization capabilities of CMR. A critical frontier is the implementation of AI-driven automated workflows, which are expected to standardize valve timing and pressure curve integration, thereby overcoming current limitations regarding inter-vendor variability and clinician workload. Standardization across vendors, refinement of disease-specific thresholds, and integration with biomarkers and advanced imaging modalities represent key priorities. Moreover, the exploration of MW in underrepresented populations—including pediatric patients and individuals with mixed valvular–myocardial disease—may broaden its clinical applicability. Ultimately, MW is poised to become a cornerstone of therapeutic guidance in structural interventions, particularly in optimizing patient selection for cardiac resynchronization therapy and transcatheter valve repairs, where quantifying myocardial efficiency is superior to conventional volumetry.

## 13. Conclusions

Noninvasive MW assessment provides a pressure-adjusted and physiologically grounded framework for evaluating LV performance, showing a promising association with clinical outcomes across diverse cardiovascular conditions. Preliminary evidence suggests that indices such as GWI and GCW may facilitate the identification of patients at increased risk of adverse events, potentially offering incremental prognostic insights in cases where conventional parameters, like LVEF and GLS, remain within borderline ranges or are influenced by loading conditions. However, given the predominantly observational nature and the inherent heterogeneity of current data, these findings should be interpreted with caution. While MW represents a robust candidate for enhanced risk stratification, its definitive role in steering clinical decision-making and its impact on long-term patient management remain to be established through large-scale, standardized prospective trials.

## Figures and Tables

**Table 1:** Main characteristics, limitations, and clinical implications of left ventricular global longitudinal strain and myocardial work [6,7].

Feature	Global Longitudinal Strain	Myocardial Work
Underlying principle	Quantification of myocardial deformation along the longitudinal axis	Integration of myocardial deformation with estimated LV pressure
Imaging technique	Speckle-tracking echocardiography	Speckle-tracking echocardiography combined with brachial cuff systolic blood pressure
Load dependency	Sensitive to afterload changes	Relatively load-adjusted by incorporating pressure estimates
Assessment of myocardial energetics	Not assessed	Provides an energetically meaningful assessment of myocardial performance
Sensitivity to early myocardial dysfunction	High	High
Performance in pressure overload conditions (e.g., hypertension, aortic stenosis)	Limited by afterload dependency	Improved evaluation by accounting for instantaneous loading conditions
Ability to differentiate acute load effects from chronic remodeling	Limited	Enhanced
Main limitations	Load dependency (particularly afterload), inter-vendor variability, and reliance on optimal image quality	Dependence on blood pressure estimation and vendor-specific algorithms; limited accounting for preload and myocardial geometry; and reliance on pressure-derived indices rather than direct measurements of myocardial force
Clinical applications	Diagnosis and risk stratification of cardiovascular diseases; established use for longitudinal surveillance of systolic function	Emerging use in risk stratification, distinguishing afterload versus contractility impairment, evaluating myocardial energetics, and predicting therapy response

**Table 2:** Normal reference ranges for healthy individuals, as reported in the EACVI NORRE study [23].

Age, Years	GWI, mmHg%		GCW, mmHg%		GWW, mmHg%		GWE, %	
	Male	Female	Male	Female	Male	Female	Male	Female
20–40	1758 ± 270	1800 ± 251	2186 ± 240	2109 ± 289	99 (68–144.5)	90 (48–145)	95 (93–97)	95 (94–97)
40–60	1900 ± 317	2027 ± 341	2267 ± 327	2329 ± 365	89 (58–122.5)	76 (51–118)	96 (95–97)	96 (95–97)
≥60	1866 ± 286	2002 ± 270	2226 ± 328	2338 ± 386	85 (49–129)	90 (48–145)	96 (94–97)	95 (94–97)

Values are reported as mean ± standard deviation or median (interquartile range).

**Table 3:** Myocardial work in cardiovascular diseases: clinical utility and key considerations.

Cardiovascular Disease	Key MW Indices	Diagnostic Utility	Prognostic Utility	Advantages	Limitations/ Considerations
Chronic coronary syndromes	GWI, GCW, GWE	Detects subclinical ischemia; outperforms GLS in patients with preserved LVEF and non-obstructive CAD; sensitive to microvascular dysfunction	Predicts high-risk CAD and adverse outcomes	Accounts for afterload; detects dysfunction despite normal LVEF	Limited applicability of a single MW index due to heterogeneity of ischemia
Acute coronary syndromes	GWE, GWI, GCW	Identifies extent of myocardial injury and regional dysfunction post-MI	Predicts mortality, LV remodeling, and MACE	Sensitive to early injury and recovery; incremental to LVEF	Timing of assessment influences interpretation
Mitral regurgitation	GWI, GCW, GWE, GWW	Identifies masked LV dysfunction despite preserved LVEF	Predicts mortality, LV remodeling, and response to TEER or surgery	Less load-dependent than LVEF/GLS; reflects true myocardial performance	Interpretation influenced by altered loading conditions
Aortic stenosis/TAVI	GWI, GCW	Detects LV dysfunction despite preserved LVEF	Predicts mortality and postoperative outcomes; superior to GLS	Incorporates pressure overload, strong prognostic value	Requires correction for transvalvular gradient
Cancer therapy-related cardiotoxicity	GWI	Identifies early dysfunction in patients with BP changes	Predicts risk of CTRCD in selected patients	Complementary to GLS; load-adjusted assessment	Incremental value mainly in selected subgroups
Hypertrophic cardiomyopathy	GCW, GWI	Reflects fibrosis burden and disease severity	Predicts adverse outcomes and arrhythmic risk	Correlates with myocardial fibrosis and hypertrophy	Limited pediatric data; not consistently superior to GLS
Dilated cardiomyopathy	GWI, GCW	Assesses impaired myocardial efficiency	Predicts mortality and HF hospitalization	Incremental value beyond LVEF and GLS	Limited accuracy due to unaccounted variations in wall thickness and ventricular geometry
Restrictive cardiomyopathy/amyloidosis	GWI, GCW, GWE, GWW	Identifies inefficient myocardial mechanics	Strong predictor of mortality and MACE	Robust prognostic performance across amyloid types	Limited availability in advanced disease stages
Heart failure	GWI, GCW, GWW	Differentiates severity and mechanical inefficiency	Predicts mortality, hospitalization, and functional decline	Applicable across HF phenotypes	Prognostic strength varies by HF stage

Abbreviations: MW, myocardial work; GWI, global work index; GCW, global constructive work; GWE, global work efficiency; GWW, global wasted work; LVEF, left ventricular ejection fraction; GLS, global longitudinal strain; CAD, coronary artery disease; MI, myocardial infarction; MACE, major adverse cardiac event; LV, left ventricle; TEER, transcatheter edge-to-edge repair; TAVI, transcatheter aortic valve implantation; BP, blood pressure; CTRCD, cancer therapy-related cardiac dysfunction; HF, heart failure.

## Data Availability

No new data were created or analyzed in this study. Data sharing is not applicable to this article.

## Abbreviations

The following abbreviations are used in this manuscript:

AS	Aortic stenosis
CAD	Coronary artery disease
CCSs	Chronic coronary syndromes
CMR	Cardiac magnetic resonance
EF	Ejection fraction
GCW	Global constructive work
GLS	Global longitudinal strain
GWE	Global work efficiency
GWI	Global work index
GWW	Global wasted work
HF	Heart failure
LV	Left ventricular
LVEF	Left ventricular ejection fraction
MACE	Major adverse cardiac events
MR	Mitral regurgitation
MW	Myocardial work
PCI	Percutaneous coronary intervention
PSL	Pressure–strain loop
PVL	Pressure–volume loop
TAVR	Transcatheter aortic valve replacement
TEER	Transcatheter edge-to-edge repair
